# Exclusion from spheroid formation identifies loss of essential cell-cell adhesion molecules in colon cancer cells

**DOI:** 10.1038/s41598-018-19384-0

**Published:** 2018-01-18

**Authors:** Mira Stadler, Martin Scherzer, Stefanie Walter, Silvio Holzner, Karoline Pudelko, Angelika Riedl, Christine Unger, Nina Kramer, Beatrix Weil, Jürgen Neesen, Markus Hengstschläger, Helmut Dolznig

**Affiliations:** 1 Institute of Medical Genetics, Medical University of Vienna, Währinger Straße 10, A-1090 Vienna, Austria; 2 Present Address: Karolinska Institutet, Solnavägen 1, 171 77 Solna, Sweden; 3 Present Address: Deutsches Krebsforschungszentrum (DKFZ), Im Neuenheimer Feld 280, 69120 Heidelberg, Germany; 4 Present Address: Boehringer Ingelheim RCV GmbH & Co KG, Vienna, Austria, Dr. Boehringer-Gasse 5-11, 1130 Vienna, Austria

## Abstract

Many cell lines derived from solid cancers can form spheroids, which recapitulate tumor cell clusters and are more representative of the *in vivo* situation than 2D cultures. During spheroid formation, a small proportion of a variety of different colon cancer cell lines did not integrate into the sphere and lost cell-cell adhesion properties. An enrichment protocol was developed to augment the proportion of these cells to 100% purity. The basis for the separation of spheroids from non-spheroid forming (NSF) cells is simple gravity-sedimentation. This protocol gives rise to sub-populations of colon cancer cells with stable loss of cell-cell adhesion. SW620 cells lacked E-cadherin, DLD-1 cells lost α-catenin and HCT116 cells lacked P-cadherin in the NSF state. Knockdown of these molecules in the corresponding spheroid-forming cells demonstrated that loss of the respective proteins were indeed responsible for the NSF phenotypes. Loss of the spheroid forming phenotype was associated with increased migration and invasion properties in all cell lines tested. Hence, we identified critical molecules involved in spheroid formation in different cancer cell lines. We present here a simple, powerful and broadly applicable method to generate new sublines of tumor cell lines to study loss of cell-cell adhesion in cancer progression.

## Introduction

The use of cancer cell lines grown on 2D plastic surfaces as a basic model to study cancer biology and a preclinical drug testing system is limited due to lack of structural architecture. 3D aggregates, known as multicellular tumor spheroids, have been developed to overcome these limitations^1^. Spheroids much better recapitulate the *in vivo* situation of tumors than cell monolayers, as they are composed of proliferating, non-proliferating, well-oxygenated, hypoxic and necrotic cells^2,3^ (reviewed in ref.^4^). Furthermore, 3D growth of cells in spheroids influences cell behavior, cell shape, polarity^5^, gene expression^6,7^, proliferation^5,7^, cell motility^8^, differentiation^9^ and drug sensitivity as well as radiation resistance^10^ (reviewed in refs^1,4^).

Multicellular spheroid formation depends on homotypic cell adhesion, which in epithelial cells is primarily mediated via the adherens junction (AJ) protein E-cadherin (CDH1)^11^. AJs are associated with the filamentous (F-) actin cytoskeleton and are crucial for epithelial sheet formation^12^. The cytoplasmic domain of classical cadherins can bind ß-catenin, which can interact via α-catenins and vinculin as well as other molecules with the actin cytoskeleton^13^. In this way, force or tension can be sensed and transduced in epithelial structures ultimately leading to altered linkage of AJs to the F-actin network^14^. E-cadherin is essential for the establishment of AJs. However, the depletion of E-cadherin in confluent epithelial sheets had little effect on the localization or function of established AJs. Differential E-cadherin expression levels have been associated with altered spheroid formation in head and neck carcinoma cell lines^15^. Differential E-cadherin expression was also associated with compact spheroid formation in hepatocellular carcinoma cell lines^16,17^ and in renal cell carcinoma^18^. In addition spheroid models were used to identify cooperative roles for E-cadherin and the desmosome proteins DSG2 and DSC2 in colon and breast carcinoma cell lines^19^. Cells lacking the linker protein α-catenin are unable to associate tightly, despite sufficient cadherin expression^20–22^. Even in established epithelial monolayers depletion of α-catenin is essential for the maintenance of AJs^23^. *In vivo*, forced E-cadherin expression represses cancer development and loss of E-cadherin^24^ or cadherin switching^25^ during epithelial to mesenchymal transition (EMT) is associated with increased mobility and tumor progression as well as metastasis^26^. Loss of α-catenin induces the formation of premalignant squamous cell cancer in skin. In addition, α-catenin acts as an invasion repressor^27^ and in colorectal cancer (CRC), reduced α-catenin levels are associated with tumor progression^28,29^.

However, this is a simplified concept focusing on two major components of the AJ complex in carcinogenesis. The reality in human cancer is by far more complex. For example in human CRC expression changes in a plethora of other cell adhesion genes have been reported, including induction of P-cadherin (CDH3)^30^, OB-cadherin (CDH11), claudins (CLDN1/2) and desmosomal proteins (DSC3, DSG2) and downregulation of CLDN5/8/23, DSC2, and protocadherins (PCDHB7/14)^31^. Interestingly, P-cadherin displayed a promotion of invasion whenever E-cadherin was also present in mammary^32,33^, pancreatic^34^ and ovarian^35^ cancer models. In contrast, in HT29 colon cancer cells loss of P-cadherin in the presence of E-cadherin induced a more invasive phenotype^30^. However, when expressed alone, P-cadherin was invariantly invasion suppressive^36–38^. In breast cancer P-cadherin overexpression is correlated with reduced tumor growth in mice and bad prognosis in human patients when E-cadherin is co-expressed in the tumor, whereas the expression of P-cadherin or E-cadherin alone displayed reduced cancer growth. Loss of both cadherins was associated with an intermediate phenotype^39^.

Thus, determining the predominant molecule mediating homotypic cell adhesion in cancer cells and loss of adhesion variants in these cells is of vital importance to study and better understand cell adhesion and cell dissemination in human cancer. Here we applied the simple method of spheroid formation to identify subclones of several spheroid-forming colon cancer cell lines, which have lost the ability to integrate into the spheroid and thus lacked the capacity of homotypic cell adhesion. These cells displayed increased migration and invasion *in vitro*. Expression profiling revealed which adhesion molecule was lost in the non-spheroid-forming (NSF) subclones. Knockdown of candidate genes in the spheroid-forming parental cells by siRNA was a fast, robust and reliable way to functionally test the involvement of the deregulated adhesion molecules in homotypic cell-cell adhesion and spheroid formation ability.

## Results

### Formation of colon cancer spheroids consistently display single cells separated from compact cell aggregates, which can be enriched by differential sedimentation

**Figure 1:**
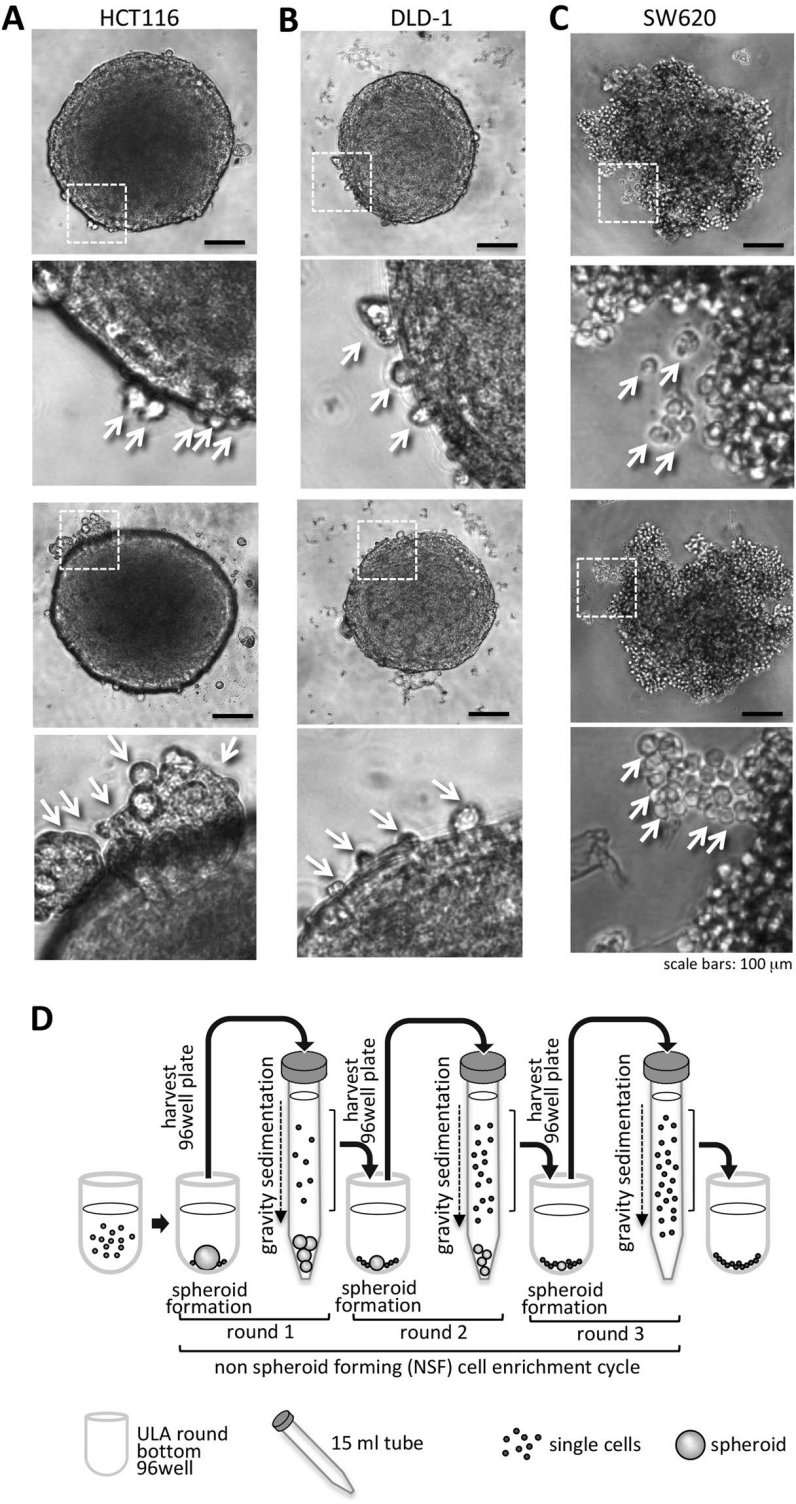
Spheroid formation as a tool to select non spheroid forming (NSF) cells. **(A)** HCT116 spheroid formation was induced by seeding tumor cells in ULA plates. After 4 days spheroids were formed from the vast majority of cells but a small amount of cancer cells were not incorporated into the compact cell aggregates (arrowheads). (**B**) DLD-1 cells were treated as in A and photographed. Non-incorporated single cells (NSF) at the periphery of the spheroid surface are indicated by arrowheads. (**C)** SW620 cells were treated as in A and photographed. Non-incorporated single cells (NSF) at the periphery of the spheroid surface are indicated by arrowheads. Scale bars in overviews: 100 µm. (**D)** Scheme of workflow to enrich NSF cells from spheroid forming (SF) ones. Spheroids were generated by seeding tumor cells into non-adhesive, U-shaped, 96-well plates. After 4 days tumor spheroids were harvested and transferred in their culture medium from 96-well plates into 15 ml centrifuge tubes. Within 3 min, gravity forced the cancer spheroids to sink to the bottom of the centrifuge tubes and the supernatant, which contained the NSF cells, was removed and further used. This procedure was repeated two times to highly enrich the NSF cells and generate stable sublines.

The colon cancer cell lines HCT116, DLD-1 and SW620 were analyzed for spheroid formation capacity in ultra low attachment (ULA) round bottom 96-well plates. We recognized that four days after tumor spheroid formation a small amount of cancer cells did not integrate into the compact spheres (Fig. 1A,B,C). These cells were visible as round, single cells at the border of the spheroids. We hypothesized that these non-spheroid forming (NSF) cells lost the ability to integrate into the spheres and aimed to identify the reason for the exclusion. For molecular analysis the enrichment and separation from the spheroid forming (SF) cells was mandatory. To augment for these NSF cells, spheroids in their culture medium were transferred into 15 ml centrifuge tubes. The spheroids were separated by three minutes of gravitational sedimentation (spheres sank to the bottom of the tube) from NSF cells, which remained - during this short time period - in the supernatant. The supernatants and pellets were collected; the cells were plated on conventional 2D culture plates and were expanded. Subsequent they were subjected for two further rounds of spheroid formation, gravity sedimentation and NSF cell harvesting procedure (Fig. 1D) to yield high purity NSF as well as SF populations, which were also used in further experiments. After three rounds of this enrichment, pure NSF and SF populations were further analyzed. Importantly, these cells displayed normal attachment to plastic surfaces in conventional 2D cultures.

### Enrichment of non-spheroid integrating cells leads to complete loss of spheroid formation capacity

**Figure 2:**
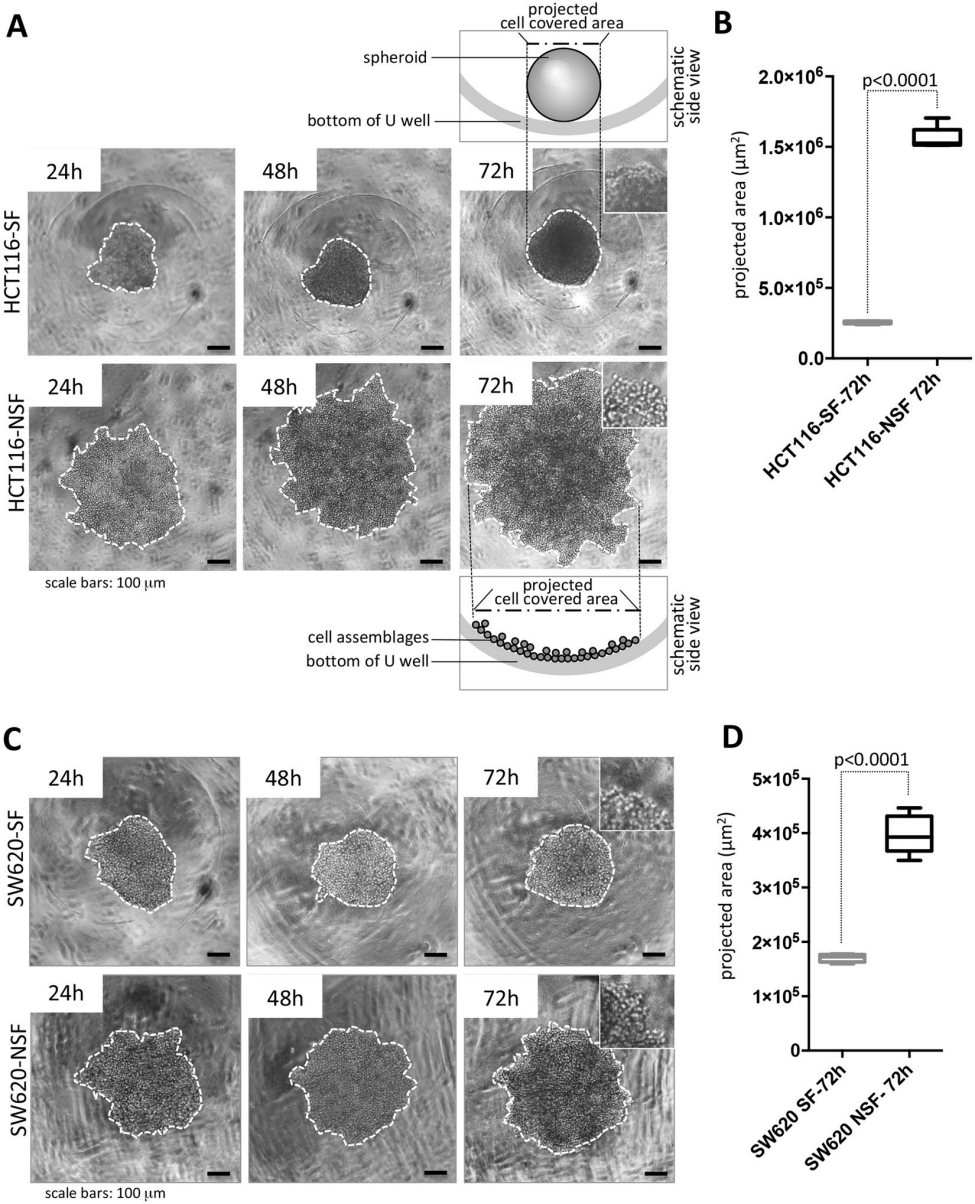
Comparison of the phenotype of HCT116-SF and SW620-SF cells versus their NSF sublines. **(A)** Time course of spheroid formation of HCT-116-SF cells compared to HCT-116-NSF. Lack of spheroid formation in the case of HCT116-NSF is evident from the formation of loosely associated cells at the base of the round well bottom. For clarity boarders of cell covered areas (either spheroid projected areas or loose cell assemblages, see schematic side view) are indicated by white dotted lines. (**B)** Quantification of projected spheroid versus cell assemblage areas of after 72 hours, n = 7 for SF and NSF each. (**C)** Time course of spheroid formation of SW620-SF cells compared to HCT116-NSF. Lack of spheroid formation in the case of SW620-NSF is evident from the formation of loosely associated cells at he base of the round well bottom. For clarity boarders of cell covered areas (either spheroids or loose cell assemblages) are indicated by white dotted lines. (**D)** Quantification of projected spheroid versus cell assemblage areas of after 72 h, n = 5 each. Boxes represent the interquartile range; the horizontal line in the box indicates the median; whiskers extend to min and max; p-values are indicated. Scale bars: 100 µm.

After the three-round separation protocol, the same number (1 × 10^3^) of HCT116-SF and HCT116-NSF cells was seeded into each well of ULA 96-well plates and cell morphology was investigated for 3 days. After 72 h, compact multicellular spheroids were formed by HCT116-SF. In contrast, HCT116-NSF cells were neither capable of producing spheroids nor displaying spheroid-like partial cell clusters after 72 h (Fig. 2A). At higher magnification it was evident that the layer of HCT116-NSF cells was composed of cells with round morphology without any obvious close cell-cell adhesion (Fig. 2A, inset). They displayed a one-cell layer appearance in the round bottom ULA 96-well plate, forced by the U-shaped form of the wells to sit next to each other. Therefore, the NSF cell-mass (expanding only in two dimensions) appeared bigger in diameter albeit the same cell numbers seeded (Fig. 2A) than the SF cells, which formed spheres (compacting and expanding in three dimensions). The cell-covered area was determined and served as a measure for spheroid formation (small area) or loose cell layers (large area). This measurement of projected cell covered areas (see schemes in Fig. 2A) was used to quantitatively describe and distinguish spheroid formation versus cell assemblage at the bottom of the U-well. The projected area was significantly increased in the NSF cells (Fig. 2B).

SW620, another colon cancer cell line, which forms more loosely aggregated spheroids (see Fig. 1A) when compared to HCT116 cells after 72 h in ULA plates were also analyzed. We wanted to investigate if the SF/NSF enrichment protocol could yield, on the one hand, more compact spheroids (SF) and, on the other hand, if it was possible to obtain NSF cells also of this cancer cell line. The aggregates generated from SW620-SF did not display any change in spheroid morphology (Fig. 2C) as compared to parental SW620. However, SW620-NSF cells showed an even looser aggregation phenotype and were present as copious sheets. These structures were composed of rounded cells without signs of tight cell-cell interaction with their neighbors (Fig. 2C). The projected cell-covered area was increased twofold in NSF vs SF (Fig. 2D). Hence, NSF subtypes can even be isolated from a cell line displaying aggregate behavior rather than spheroid appearance.

### The NSF phenotype is reproducibly generated in DLD-1 and SW620 colon cancer cells

**Figure 3:**
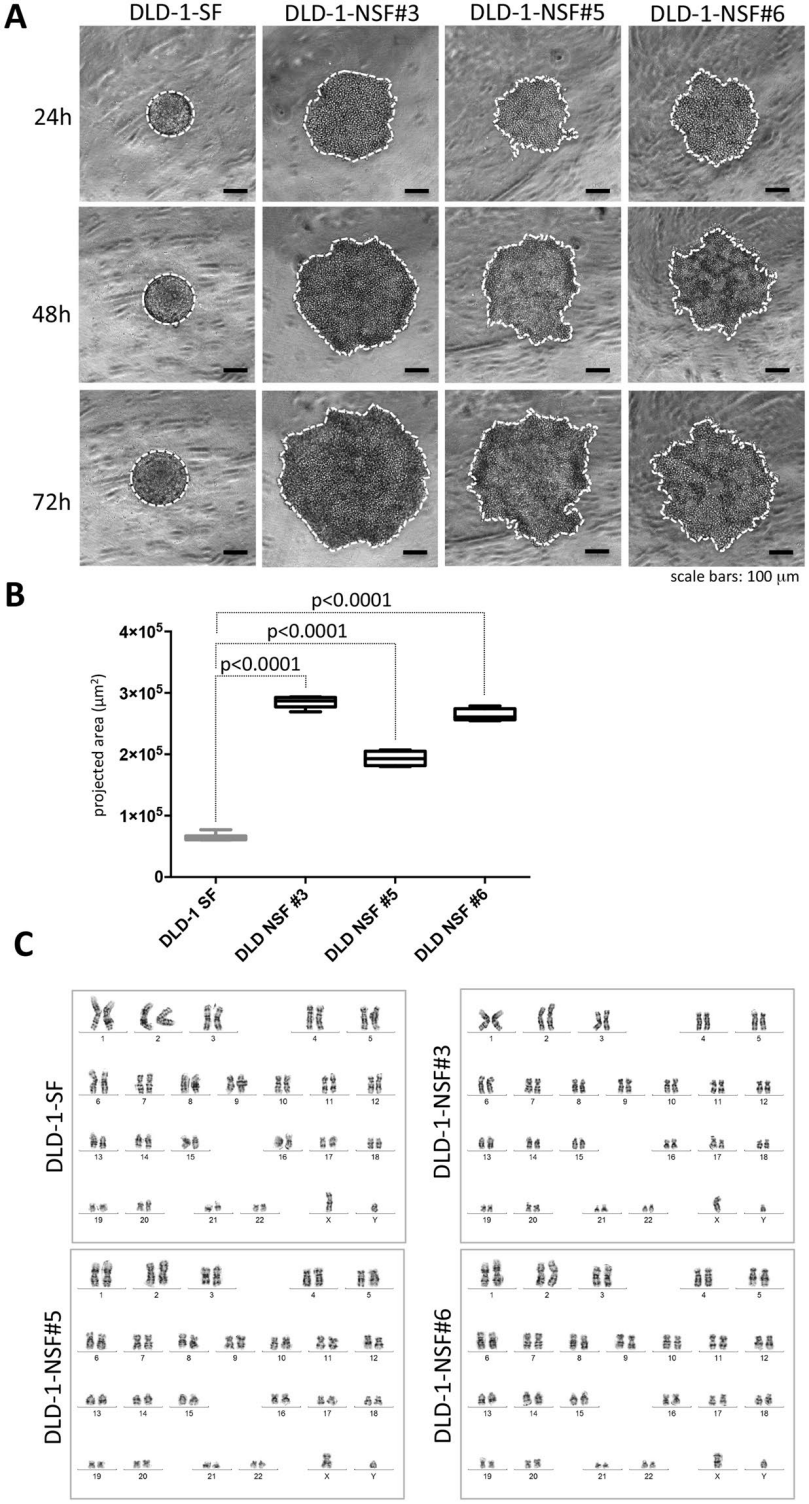
DLD-1-NSF cells can be reliably obtained and display no chromosomal aberrations compared to their parental origin. **(A)** DLD-1 cells were seeded into ULA round bottom 96-well plates. After 2 weeks spheroids were separated from non-integrated NSF-cells by sedimentation (see Supplemental Figure S1), cultured in 2D and 6 individual DLD-1-NSF sublines were generated. DLD-1-NSF sublines were analyzed for spheroid formation capacity. Representative images of DLD-1 SF and DLD-1 NSF sublines #3, #5 and #6 after 24 hours, 48 hours and 72 hours at spheroid inducing conditions. Scale bars: 100 µm. (**B)** Quantification of projected spheroid versus cell assemblage areas of after 72 hours (cell covered areas), DLD-1 SF n = 6, DLD-NSF #3 n = 5, #5 n = 4, #6 n = 4. Boxes represent the interquartile range; the horizontal line in the box indicates the median; whiskers extend to min and max. P-values are indicated. (**C**) Karyogramms of DLD-1-SF and NSF sublines #3, #5, #6.

Next, we examined the reproducibility of NSF formation using the above-described SW620 and DLD-1 cells. We aimed to collect individual DLD-1-NSF and SW620-NSF sublines from single wells of ULA round bottom 96-well plates. For this, 2 × 10^2^ DLD-1 or SW620 cells were seeded into each well and were subjected to long-term culture (2 weeks) with partial medium changes every third day to supply the spheres with fresh medium. After 14 days of expansion spheroids were grown and interestingly non-integrated NSF cells were also significantly expanded in number (Supplemental Figure S1A). Spheroids were separated from single cells by sedimentation in microcentrifuge tubes and cells were expanded in 2D without further selection. Four SW620 (Supplemental Figure S1B) and six DLD-1 single cell phenotype sub-lines were further analyzed. All of them stably displayed the loss of spheroid formation phenotype (three of the DLD-1 sublines are shown in Fig. 3A) and kept it also after several freeze and thaw cycles. This was also quantitatively verified by assessing the cell-covered area (Fig. 3B).

The near diploid chromosome set of parental DLD-1 was retained in all DLD-1-NSF sub-lines, as shown by lack of aberrations in metaphase chromosome spreads (Fig. 3C). This indicates that despite the microsatellite instability (MSI) phenotype of DLD-1 there is, at the chromosome level, no selection for grossly aberrant clones by the selection procedure.

### The NSF phenotype is associated with increased migratory potential and augmented invasion

**Figure 4:**
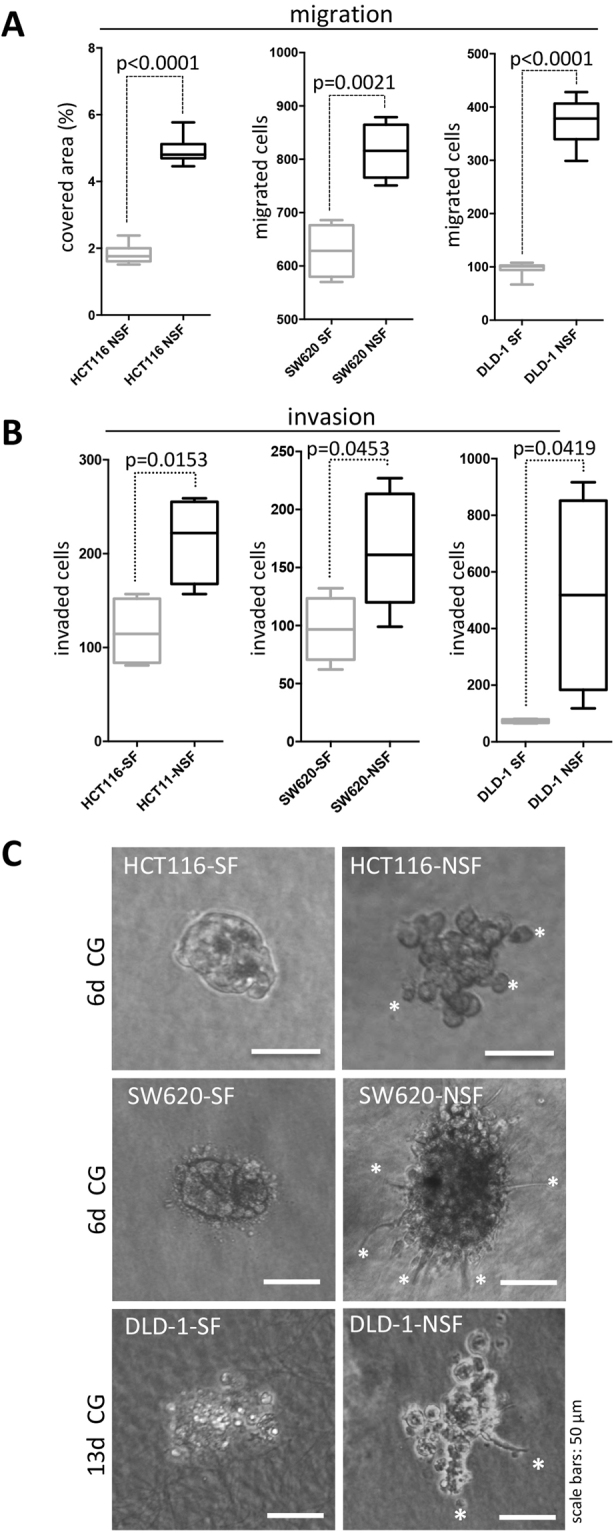
Increased migration and invasion of NSF sublines. **(A)** For assessment of migration a transwell migration assay was performed and the number of migrated cells was determined after 20 h. In case of HCT116 and DLD-1 cells eight transwell inserts were evaluated; for SW620 cells n = 4. (**B)** A transwell invasion assay was used and cells invaded through collagen I covered membranes were counted after 24 hours; n = 4 for each individual experiment. Boxes represent the interquartile range; the horizontal line in the box indicates the median; whiskers extend to min and max. P values are indicated. (**C)** Representative phase contrast pictures of colonies grown from single cells seeded into collagen I gels (CG). Pictures were taken after 6 days (HCT116 and SW620 lines) and 13 days (DLD-1 lines). White asterisks indicate single cells or invasive structures protruding into the matrix. Scale bars: 50 µm.

In a further set of experiments, we asked whether the loss of the spheroid formation phenotype resulted in further phenotypic differences. In all three lines cell migration was significantly increased in the NSF setting. DLD-1-NSF, HCT116-NSF and SW620-NSF displayed a fourfold, 2.5 fold and 1.3 fold higher migratory ability, respectively, than their corresponding SF counterparts (Fig. 4A). Similarly, invasion through a collagen I matrix was significantly elevated in all three NSF cultures as compared to their corresponding SF sublines (Fig. 4B). Moreover, when seeded as single cells in a collagen I gel, HCT116-SF and SW620-SF formed small compact round colonies after 6 days of culture. In sharp contrast, HCT116-NSF displayed loosely, irregularly formed colonies with cells protruding into the gel matrix. SW620-NSF produced larger colonies than the SF cells and showed spindle like cells invading into the collagen gel (Fig. 4C, Supplemental Figure S2A, B). Interestingly, DLD-1-SF cells died when seeded as single cells into the matrix, whereas DLD-1-NSF displayed irregular colonies with rounded cells and occasional spindle like protrusions (Fig. 4C, Supplemental Figure S2C).

### Quantitative PCR array expression profiling identifies loss of molecules involved in cell adhesion in SW620-NSF, HCT116-NSF and DLD-1-NSF cells

**Figure 5:**
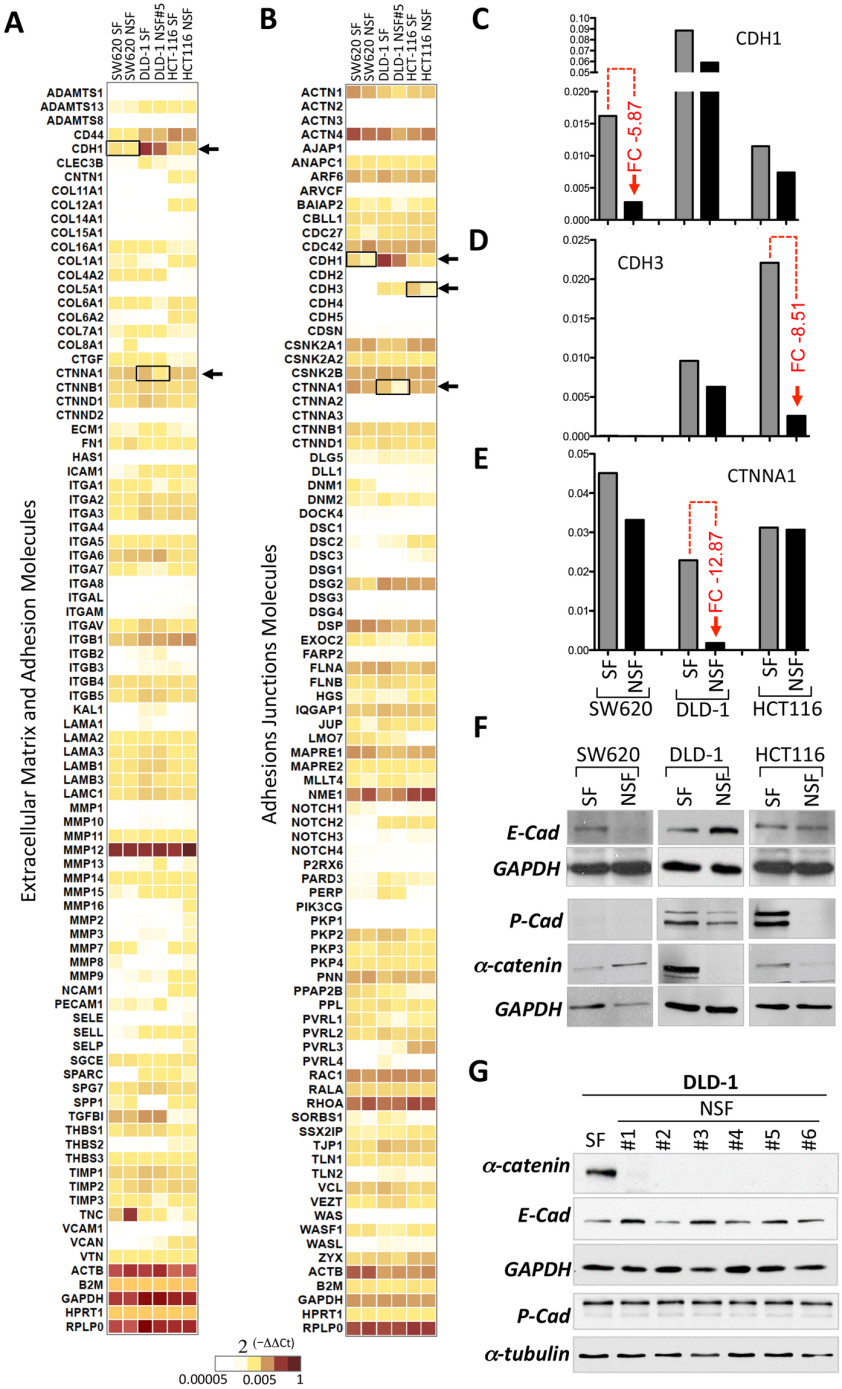
Detection of molecular changes in NSF cells versus SF cells using RT2 profiler qPCR arrays. **(A)** SW620, DLD-1 and HCT116 SF-and NSF-cells were subjected to sphere forming conditions and mRNA was isolated and analyzed with RT^2^ PCR profiling to obtain expression patterns for 160 adherens junction, extracellular matrix and adhesion molecules. Heat maps indicating the level of expression representing are shown (red: high expression, yellow: intermediate expression, white low or absent). Full dataset available in Supplementary Table 1. (**C–E)** Quantification of CDH1, CDH3 and CTNNA1 mRNA expression of the data from (**A**). Maximal fold changes (FC) between SF and NSF are indicated in red. (**F)** E-(CDH1), P-cadherin (CDH3) and α-catenin (CTNNA1) protein expression was assessed by Western blotting. GAPDH is shown as loading control. (**G)** Western blot analysis of α-catenin as well as E- and P-cadherin expression in the DLD1-NSF subclones #1–6. GAPDH and α-tubulin are shown as controls.

Using two qPCR arrays (RT2 profiler, Qiagen) we aimed to identify the molecular reasons for loss of cell-cell adhesion in the NSF sublines of DLD-1, HCT116 and SW620 cells. These arrays revealed the mRNA expression levels of 162 cell-to-cell and cell-to-matrix adhesion genes and adhesion-related molecules in SW620-SF, SW620-NSF, DLD-1-SF, DLD-1-NSF and HCT116-SF as well as HCT116-NSF (Heatmaps, Fig. 5A,B; full dataset in Supplementary Table 1). The mRNAs most effectively lost in SW620-NSF, HCT116-NSF and DLD-1-NSF compared to their respective SF counterparts were E-cadherin (CDH1), P-cadherin (CDH3) and α-catenin (CTNNA1), respectively; see Fig. 5A–E. A complete list of all genes analyzed including Ct values, negative and positive controls as well as fold changes are given in Supplementary Table 1. Loss of these molecules in the NSF cells was confirmed at the protein level in Western blot experiments. E-cadherin was lost only in SW620-NSF but was maintained in the other NSF lines, whereas HCT116-NSF showed selective lack of P-cadherin and DLD-1-NSF displayed unique loss of expression of α-catenin (Fig. 5F). This lack of α-catenin was consistently seen in any individually generated DLD-1-NSF subline, whereas E- and P-cadherin displayed constant high expression (Fig. 5G).

### Lack of cell-cell adhesion by loss of one adhesion protein in the NSF phenotype is not associated with changes in expression and distribution of other adhesion markers

**Figure 6:**
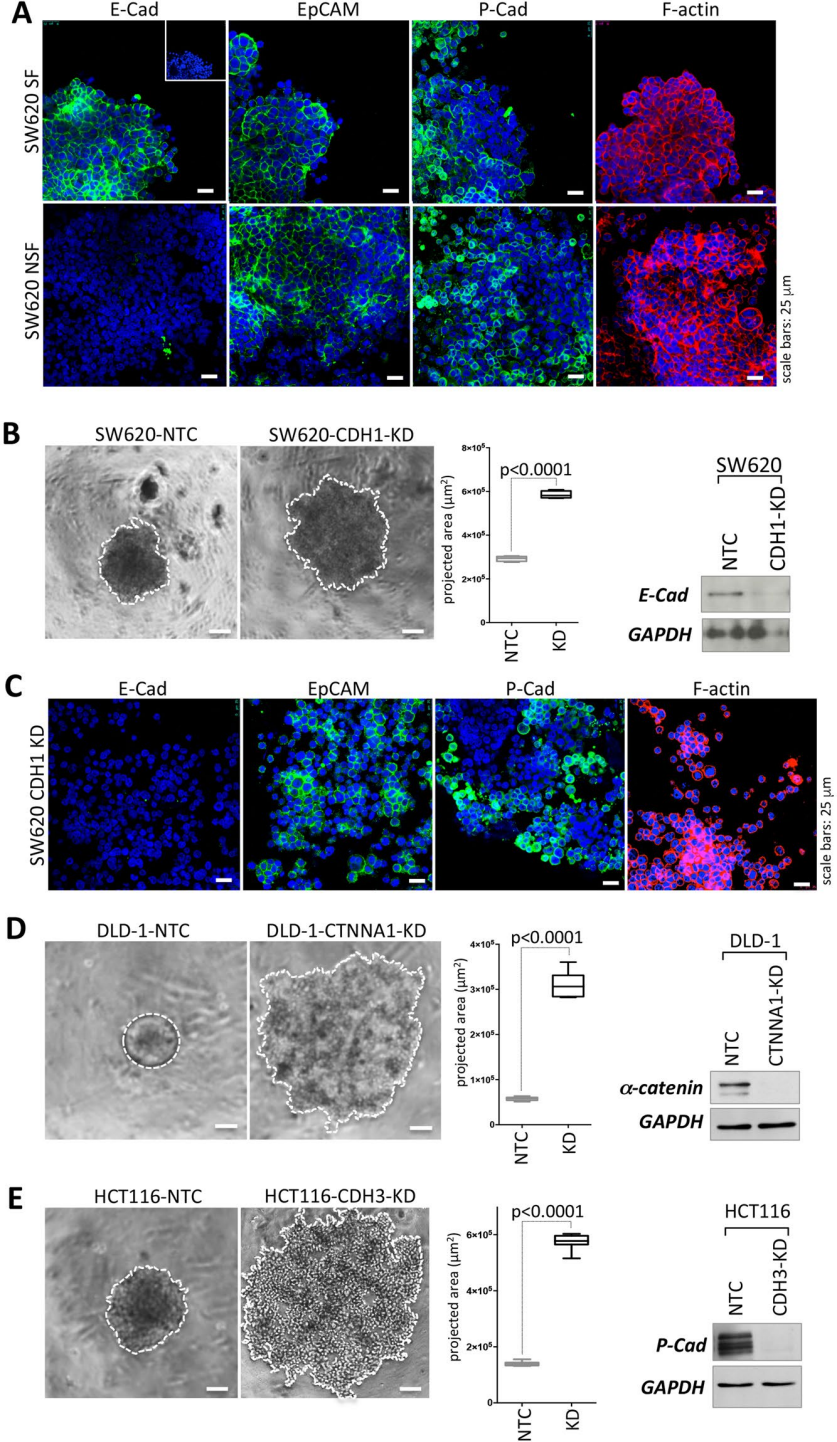
Distribution of adhesion markers and functional assessment of loss of E-cadherin, P-cadherin and α-catenin for spheroid formation by RNA interference. (**A**) IF analysis of E-cadherin, EpCAM, P-cadherin (all green) and F-actin (red) expression in SW620-SF and SW620-NSF. Cell nuclei are stained with DAPI (blue). Small inset: IgG control. Scale bars: 25 µm. B left panel, representative phase contrast micrographs of SW620 NTC vs SW620-CDH1-KD. Scale bars: 100 µm. (**C**) IF analysis of E-cadherin, EpCAM, P-cadherin (all green) and F-actin (red) expression in SW620-NTC and SW620-CDH1 KD cells. Cell nuclei are stained with DAPI (blue) Scale bars: 25 µm. Representative phase contrast pictures of (**D**) DLD-1-NTC vs DLD-1-CTNNA-KD, (**E**) HCT116-NTC vs HCT-CDH3KD (left panels). Cell covered areas are encircled with a white dotted line for clarity. Scale bars: 100 µm. (**B**,**D**,**E**) Middle panels Quantification of spheroid versus cell assemblage areas after 72 h (projected areas), SW620 n = 4 each; DLD-1-NTC n = 9, DLD-1-CTNNA1-KD n = 8; HCT116, n = 8 each. Boxes represent the interquartile range; the horizontal line in the box indicates the median; whiskers extend to min and max. P-values are indicated. (**B**,**D**,**E**) right panels Knockdown efficiency was determined by Western blotting.

The single loss of either E-cadherin, P-cadherin or α-catenin could potentially effect the expression or (sub)cellular distribution of other important adhesion molecules. To test this, spheroids were immunofluorescently stained with the cell adhesion markers E-cadherin, P-cadherin, epithelial cell adhesion molecule (EpCAM) and F-actin (Fig. 5A) and optical section were recorded with a confocal microscope. Loss of E-cadherin was evident in SW620-NSF cells (Fig. 6A) as already seen in Western blots (compare Fig. 5F), but did not affect EpCAM, P-cadherin of F-actin expression or distribution (Fig. 6A, Supplemental Figure S3). The same was evident when DLD-1-SF and DLD-1-NSF cells (Supplemental Figure S4) as well as HCT116-SF and HCT116-NSF cells were analyzed (Supplemental Figure S5). Taken together, these data imply that no gross alterations of other cell adhesion molecules were induced upon the loss of one specific molecule in the distinct cell types.

### Knockdown of E-cadherin, P-cadherin or α-catenin lead to a NSF phenotype in SW620, HCT116 or DLD-1, respectively

Next we tested, whether loss of the respective molecules identified above in the NSF colon cancer cells were causally responsible for the lack of cell adhesion during spheroid formation. For this the molecules were knocked down by siRNA in the corresponding parental cell lines. Indeed loss of E-cadherin in SW620 cells (SW620-CDH1-KD) phenocopied the SW620-NSF cells in respect to the incapability of spheroid formation, whereas non-targeting controls (SW620-NTC) displayed proper cell aggregation like parental SW620 (Fig. 6B, compare to Fig. 2A). In accordance to the loss of E-cadherin in the NSF cells, CDH1 knockdown in NTC siRNA transfected parental SW620 cells did not change levels or localization of other junction proteins like P-cadherin, EpCAM or the junction associated protein F-actin as demonstrated by immunofluorescence analysis (Fig. 6C, Supplemental Figure S6).

Similarly, DLD-1-CTNNA1-KD (Fig. 6D) and HCT116-CDH3-KD (Fig. 6E) lost spheroid formation ability. Strikingly, these knockdown cells displayed exactly the same phenotype as the respective NSF sublines (compare to Figs 2A and 3A). NTC controls formed spheres indistinguishable from non-modified DLD-1 or HCT116 cells. High knockdown efficiency was verified by Western blotting (Fig. 6B–D, right panel).

### Functional assessment of loss of E-cadherin, P-cadherin and α-catenin for spheroid formation

**Figure 7:**
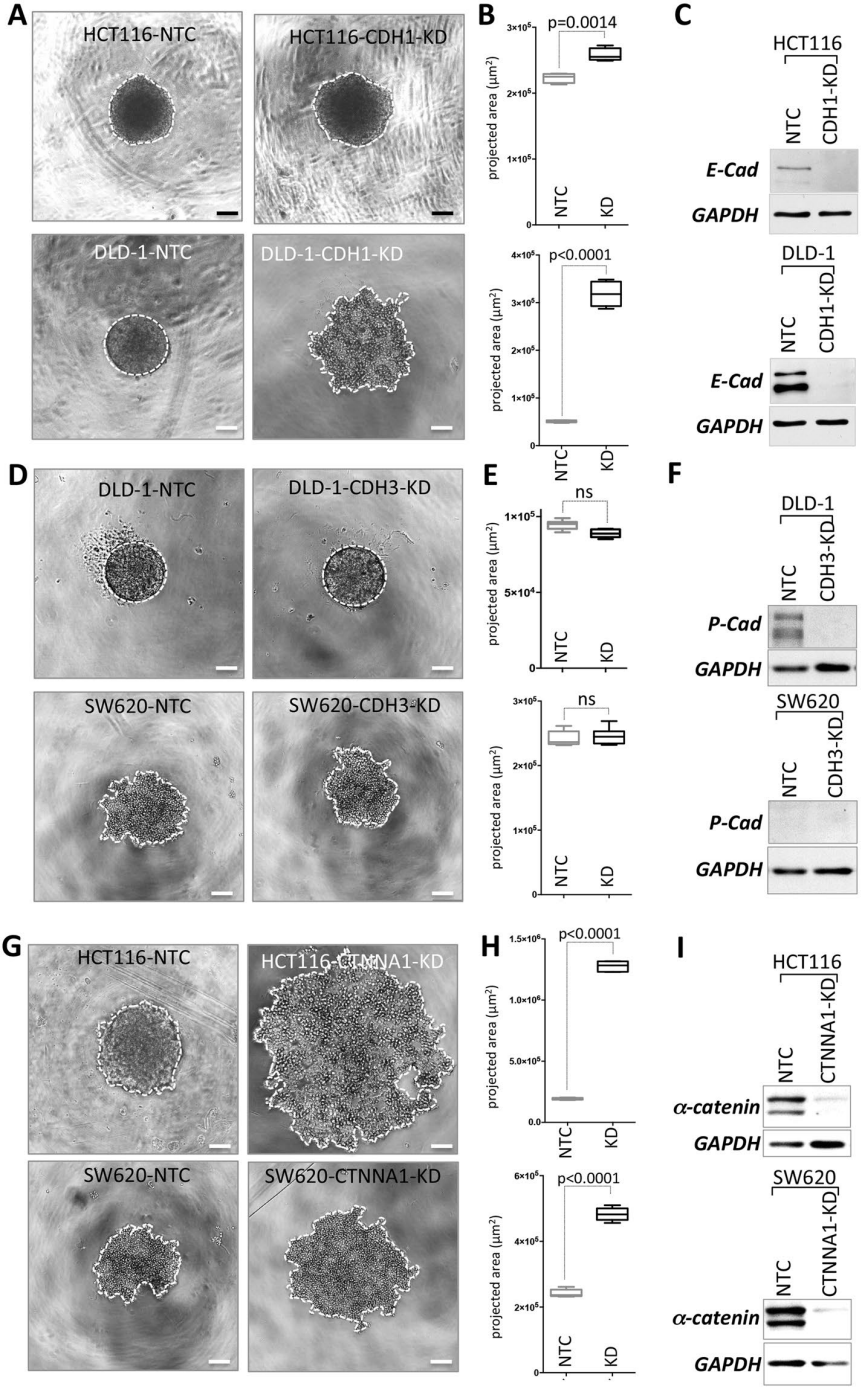
Phenotypes induced by loss of E-cadherin, P-cadherin and α-catenin by RNA interference. (**A**,**D**,**G)** Phase contrast micrographs of cells cultivated under spheroid forming conditions for 48 hours. Cell covered areas are encircled with a white dotted line for clarity. Scale bars: 100 µm. **(A)** CDH1 knockdown in HCT116 and DLD-1 cells. (**D)** CDH3 knockdown in DLD-1 and SW620 cells. (**G)** CTNNA1 knockdown in HCT116 and SW620 cells. (**B,E,H)** Quantification of projected spheroid versus cell assemblage areas of after 48 hours (cell covered areas). Boxes represent the interquartile range; the horizontal line in the box indicates the median; whiskers extend to min and max. P-values are indicated and non-significant comparisons are labeled with ns. (**B**) HCT116-NTC, HCT116-CDH1-KD n = 4 each; DLD-1-NTC, DLD-1-CDH1-KD n = 6 each; **E** DLD-1-NTC, DLD-1-CDH3-KD n = 6 each; SW620-NTC, SW620-CDH3-KD n = 6 each. H HCT116-NTC, HCT116-CTNNA1-KD n = 6 each; SW620-NTC n = 6, SW620-CTNNA1-KD n = 5. (**C,F,I**) Knockdown efficiency was determined by Western blotting.

E-cadherin is the major homotypic cell-cell adhesion molecule for epithelial cells and it was expressed at comparable levels in all three colon cancer cell lines analyzed. However, downregulation was only seen in SW620-NSF cells. Therefore, we were interested, whether forced loss of E-cadherin expression in HCT116 and DLD-1 cells would interfere with spheroid formation. Interestingly, in HCT116 cells knockdown of E-cadherin (CDH1) did not disrupt spheroid establishment at all (Fig. 7A), whereas in DLD-1 loss of E-cadherin impaired spheroid formation (Fig. 7B). In both cell types E-cadherin was efficiently reduced at the protein level (Fig. 7C). In two other colon cancer cell lines (LS174T and HT29), which were not included in the initial study, ablation of E-cadherin expression also induced a NSF phenotype (Supplemental Figure S7).

DLD-1 cells expressed also high levels of P-cadherin (CDH3) in addition to E-cadherin (see Fig. 5F). Therefore, we were interested whether depletion of P-cadherin might have an effect on proper spheroid formation in these cells. Again, siRNA-mediated knockdown of CDH3 was performed in parental DLD-1 cells and spheroid formation was monitored. No difference in compact spheroid formation was detectable between DLD-1-NTC and DLD-1-CDH3 (Fig. 7D,E), in spite of highly effective knockdown as verified by almost complete loss of P-cadherin protein expression in the siRNA treated cells (Fig. 7F). As SW620 cells did not express P-cadherin (see Figs 4 and 7F) no change of phenotype was expected when these cells were treated with CDH3 siRNA. Indeed, there was no difference in spheroid formation detectable and NTC and CDH3 siRNA transfection of SW620-SF cells led to the same spheroid formation phenotype as seen in parental SW620-SF cells (Fig. 7D,E). This experiment also demonstrated that the transfection approach with a targeting siRNA *per se*, did not alter the spheroid formation ability.

Due to its proposed central mechanical role as a tension transducer in the development of adherens junctions^14^ and its essential role for cell adhesion^20–22,40,41^ we speculated that α-catenin (CTNNA1) might have a major role in spheroid formation in all our models. Indeed, knockdown of α-catenin in all three cell lines tested (DLD-1, SW620 and HCT116) completely impaired spheroid formation (Figs 6D and 7G,H and Supplemental Figure S8). Depletion of CTNNA1 was highly efficient at the protein level (Figs 6D and 7I).

### Double knockdown of E- and P-cadherin results in a severe loss of cell adhesion phenotype in DLD-1 but not in HCT116

Next, we examined whether in HCT116 and DLD-1, which express E-cadherin and P-cadherin simultaneously, double knockdown of both cell adhesion molecules would result in a more severe phenotype. This would suggest that both adhesion molecules are participating in cell-cell adhesion in these cells leading to residual cell to cell binding activity, if only one is knocked out. Results from above indicated that E-cadherin is the major factor mediating strong cell-cell adhesion and spheroid formation in DLD-1, whereas in HCT116 P-cadherin has this function.

**Figure 8:**
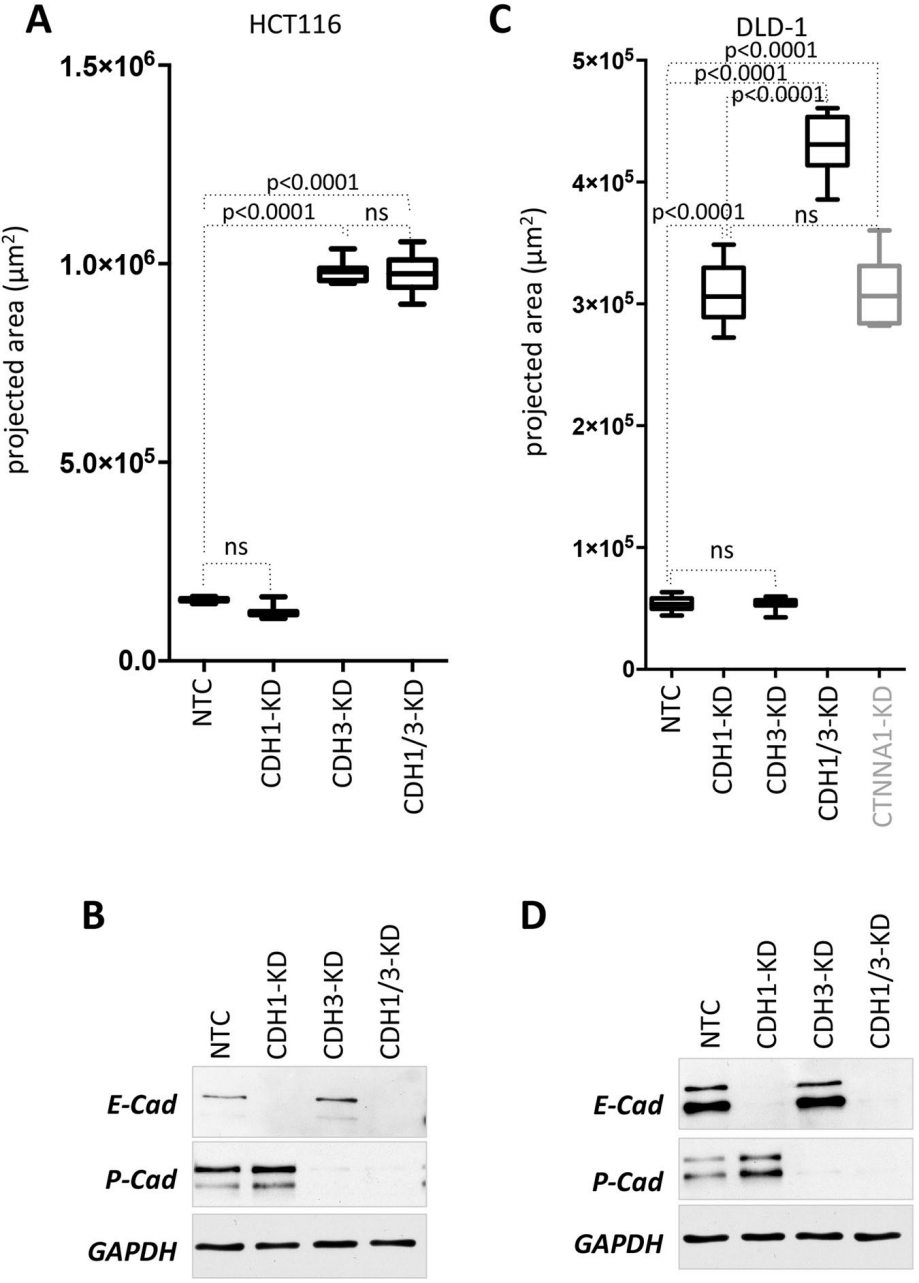
Effect of double versus single knockdowns in HCT116 and DLD-1 cells. (**A,C)** Quantification of projected spheroid versus cell assemblage areas of after 48 h (cell covered areas). Boxes represent the interquartile range; the horizontal line in the box indicates the median; whiskers extend to min and max. P-values are indicated and non significant comparisons are labeled with ns. HCT116-NTC (n = 12), HCT116-CDH1-KD (n = 12); HCT116-CDH3-KD (n = 10), HCT116-CDH1/3-KD (n = 13); DLD-1-NTC (n = 17), DLD-1-CDH1-KD (n = 12), DLD-1-CDH3-KD (n = 15), DLD-1-CDH1/3-KD (n = 17), DLD-1-CTNNA1-KD (n = 8). (**B,D**) Knockdown efficiency was determined by Western blotting.

In HCT116 double knockdown of CDH1 and CDH3 had no surplus effect on the phenotype compared to HCT116-CDH3 KD. In relation to HCT116-NTC, the increase in cell-covered area remained the same in HCT116-CDH1-CDH3-KD as compared to HCT116-CDH3-KD (Fig. 8A). Of note, E-cadherin knockdown alone again had no effect on spheroid formation at all. All molecules were efficiently depleted in the respective knockdowns or knockdown combinations as demonstrated by Western blotting (Fig. 8B).

Interestingly, in DLD-1, double knockdown of CDH1 and CDH3 resulted in significantly increased cell-covered areas as compared to DLD-1-CDH1-KD (Fig. 8C). This indicated that, when E-cadherin is lost, there is still residual low cell-cell binding capacity present, which is mediated by P-cadherin in these cells. Surprisingly, depletion of α-catenin resulted in a phenotype similar to E-cadherin loss alone (Fig. 8C,right panel), suggesting that unexpectedly residual P-cadherin mediated residual cell adhesion might be independent of α-catenin. Again, proper knockdown was verified by Western blotting (Fig. 8D).

## Discussion

Cancer progression is often associated with a change of cell adhesion properties such as loss of tight cell-cell interaction^24^, cadherin switching during EMT^25^, loss of E-cadherin or induction of cell adhesion molecules being not expressed in the normal epithelial cells of the corresponding tissue^31,42^. We report here a simple, rapid and cost efficient way to select for cancer cells with diminished homotypic cell-cell interaction. The exclusion of subpopulations of cells into multicellular spheroids could be used as a highly reproducible and robust way to select for loss of cell adhesion variants in the allegedly homogenous cell population of various established colon cancer cell lines (HCT116, DLD-1, SW620). Of note, this selection procedure was applicable in cell lines displaying a wide range of different spheroid phenotypes. Spheroid morphology ranged from tightly associated, compact and dense spheroids with established tight junctions (DLD-1^43^) via an intermediate phenotype (HCT116) to rather loose cell aggregates (SW620). Despite this variation, the gravity based selection process (see Fig. 1C) was effective to produce non spheroid forming (NSF) sublines from these three cancer cell lines. There are several ways of spheroid formation (ultralow attachment plates, presence of methylcellulose to avoid attachment of the cells to plastic, hanging drops or specialized plates for hanging drop like spheroid formation for example from InSphero) or stirring. These are different technical approaches to obtain the same principle: loss of attachment to the culture plate and therefore induction of cell-cell adhesion and spheroid formation. For isolating NSF cells by gravity all these methods would be feasible, however, a 96-well round bottom setting is essential for visual inspection of the appearance of NSF cells nearby the spheroid.

Moreover, as demonstrated for DLD-1 and SW620, parallel selection rounds always gave rise to multiple NSF sublines (Supplemental Figure S1) which displayed no changes in chromosome content (see Fig. 3) compared to the parental cells. These independently generated NSF sublines all displayed the same NSF phenotype and at the molecular level consistently lost α-catenin expression, whereas E- and P-cadherin levels did not change. Thus, these results implicate that the enrichment protocol for NSF cells might be a widely applicable and consistent novel method to isolate loss of cell adhesion mutants from other cancer cell lines of different origin or presumably also from primary patient material when grown as cancer fragment explants^44^ or as organoids^45^.

Molecular profiling of the NSF population and comparison to the parental or SF counterpart using qPCR profiling (as done in our approach with RT2 profiler PCR arrays for cell junction and adhesion molecules) readily identified candidate genes, which were significantly different in the SF vs NSF variants. More global analysis by full genome profiling (arrays or RNAseq) would be another possibility. Of note, the majority of these genes were not regulated, indicating that the protocol did not select for or even induced grossly abnormal cancer cell subtypes but rather identified highly specific changes in the NSF sublines. This was further supported by the unchanged DLD-1 karyotype in the NSF versus the parental cells as discussed above.

The identification of target genes allowed rapid functional testing by siRNA-mediated knockdown with an easy and fast readout. For this, selective knockdown of the identified target in the parental (i.e. spheroid forming) population and subsequent culture of these cells under spheroid forming conditions is performed. If the identified target is indeed responsible for homotypic cell-cell adhesion mediated spheroid formation, the knockdown cells are no longer able to form spheroids as compared to cells transfected with non-targeting control siRNA. Thus, simple functional experiments with a highly sensitive biological readout (spheroid formation versus loss of spheroid formation) rapidly validated identified target genes. In our experiments we identified three different molecules being responsible for the loss of spheroid formation ability in three different colon cancer cell sublines, respectively. This mechanistic testing demonstrated that in SW620-NSF loss of E-cadherin was responsible for the inability to form spheroids, whereas in HCT116-NSF lack of P-cadherin and the absence of α-catenin in DLD-1-NSF were the reasons for impaired spheroid formation.

HCT116 cells express both E-cadherin and P-cadherin proteins in parallel (see Figs 5F and 7C,I). In HCT116 a frameshift mutation of CDH1 (c.357delG, p.H121fs*94) at one allele has been reported by Efstathiou *et al*.^46^ and Cosmic database mining with the CanSAR website^47^, which leads to heterozygous deletion of the majority of the E-Cadherin protein encoded by this allele. The other allele is unaffected and full length E-cadherin is detected in Western blots and is located at cell-cell contacts (our results and Tay *et al*.^48^). Whether the heterozygous mutation leads to a dominant negative form has not been investigated so far. However, we show here for the first time that HCT116 spheroid formation is independent from E-cadherin expression but critically dependent on the presence of P-cadherin. Of note, P-cadherin expression in HCT116 is known and a specific antibody against P-cadherin (PF-03732010) disrupts cell aggregate formation in HCT116 and has anti-metastatic effects *in vivo* in mice HCT116 xenograft experiments^49^. Thus, we conclude that in HCT116 a subpopulation is steadily emanating that loses P-cadherin expression leading to the loss of cell-cell adhesion phenotype. In line with this, even the selected SF sublines of HCT116 produce NSF cells. The molecular reason for the P-cadherin loss is not known so far and additional experiments are necessary to further evaluate the phenotype *in vitro* and *in vivo*.

In contrast, in DLD-1 forced depletion of E-cadherin but not P-cadherin resulted in the loss of spheroid formation. However, loss of E-cadherin was not detected in the naturally occurring NSF variants isolated by the NSF selection protocol despite analysis of seven independent attempts. In DLD-1 α-catenin was consistently lost in the NSF subclones. Natural round-shaped variants of DLD-1 cells deficient for α-catenin were reported earlier and these cells also displayed impaired cell aggregation ability^21^. Cell-cell adhesion could be restored by re-expressing wildtype α-catenin in these cells^50^ leading to decreased proliferation in 3D. Strikingly, loss of α-catenin was shown for a subpopulation of HCT-8 cells, which displayed a round morphology phenotype^28^. The colon cancer cell line HCT-8 derived from the same patient and is identical to DLD-1^27^ as well as HCT-15 and HRT-18^51^. This was further validated by STR profiling, RNAseq, mutational analysis and drug response pattern^52^. The CTNNA1 gene is heterozygously mutated in DLD-1/HCT-8/HCT-15/HRT-18. Due to genetic instability as a consequence of a mutation in the HMSH6 mismatch repair gene, round-shaped cell variants occur spontaneously, all carrying either a mutation or exon skipping in the second CTNNA1 allele^27^. These mutants lacking α-catenin expression were shown to be more invasive in a chick heart invasion assay^27^. Thus, these data clearly demonstrate that two totally different assays based on phenotypic appearance (round appearance versus exclusion from spheroid formation) could identify the same mutant subpopulations of cells. The spheroid assay might be of advantage for high throughput screening to identify loss of cell-cell adhesion in any parental spheroid-forming cell line and less experienced researchers in cell biology might find it easier to identify and isolate such variants by the spheroid assay than by assessing rather subtle morphological differences in 2D culture.

Interestingly, despite the reported dysfunctional mismatch repair in DLD-1, we could not identify lack of E-cadherin expression in any of the DLD-1-NSF sublines; it was always α-catenin affected. However, artificial depletion of E-cadherin by siRNA-mediated knockdown displayed the same NSF phenotype as loss or mutation of CTNNA1. Either depletion of E-cadherin or α-catenin led to the very same phenotype and the cell-covered area was indistinguishable under non-attachment conditions. Loss of P-cadherin in DLD-1 did not disrupt the spheroid forming ability at all. Unexpectedly however, depletion of E-cadherin and P-cadherin by double knockdown resulted in an even stronger loss of cell-cell adhesion, and a significantly increased cell-covered area than NSF phenotypes induced by E-cadherin or α-catenin downmodulation alone (see Fig. 7). This was not the case for HCT116 cells, indicating a specific effect. We speculate that loss of both E- and P-cadherin result in total loss of cell adhesion, whereas there is obviously a residual weak cell adhesion activity left in cells devoid of either E-cadherin or α-catenin, and which is facilitated by P-cadherin. Thus, P-cadherin is able to mediate a weak but measurable cell-cell adhesion effect, which is clearly independent of α-catenin.

Taken together, on the one hand we provide here a novel protocol to conveniently and reliably isolate subpopulations of cancer cell lines with altered cell-cell adhesion properties leading to a non-spheroid forming phenotype. On the other hand, we identified the molecular reason for this loss of adhesion in three colon cancer cell lines using expression profiling and functional testing with a siRNA approach. This is valid to all cell lines of different cancers with spheroid forming capacity. Thus, as changes in cell-cell adhesion properties of cancer cells are critically involved in cancer progression and dissemination, our method provides a broadly applicable protocol and our results on the molecular reasons for the loss of homotypic cancer cell adhesion build the basis for further studies on cell adhesion phenotypes and will shed new light on the impact of these changes on cancer cell growth, invasion and metastasis.

## Methods

### Cell culture

Human colon cancer cell lines DLD-1 (ATCC®# CCL-221TM), HCT116 (ATCC®# CCL-247 TM) and SW620 (ATCC®#CCL-227 TM) were obtained from the American Type Culture Collection (ATCC® Rockville, MD, USA) and were cultured in Dulbecco’s modified Eagle’s medium (DMEM), high glucose (4.5 g/l), or in RPMI respectively, supplemented with 10% fetal calf serum, 2 mM L-Glutamine and antibiotics (60 mg/l penicillin, 100 mg/l streptomycin sulfate) at 37 °C and 5% CO2.

### Spheroid formation

Spheroid formation was induced as described^53^. In brief, 3000 colon cancer cells were seeded in 100 µl cell culture medium per well in an ultra-low attachment (ULA) 96-well plates (Nunclon Sphera, round bottom, Thermo Scientific). The plates were centrifuged for 15 min at 300 g in order to ensure immediate close contact of cells and thereafter spheroids were formed for 3 days and grown in DMEM high glucose (4.5 g/l), supplemented with 5% fetal calf serum, 2 mM L-Glutamine and antibiotics (60 mg/l penicillin, 100 mg/l streptomycin sulfate). Centrifugation is not essential for spheroid formation but mediates more rapid spheroid formation.

### Metaphase Spreads (Karyogram analysis)

Metaphase preparation was carried out by standard methods. By adding 0.1 μg/ml colcemid (Gibco, ThermoFisher) dividing cells were locked in metaphase for 60 minutes. Then cells were treated for 15 minutes with hypotonic solution and fixed using methanol and acetic acid. Afterwards cells were dropped onto slides, dried at 42 °C for 30 minutes and then incubated at 60 °C over night. Slides were used for Giemsa-trypsin banding of chromosomes. Karyotyping was done using the “MetaSystems Ikaros” software version 5.3.18.

### Expression profiling using RT2 profiler arrays

For gene expression analysis, ‘Human Adherens Junctions’ and ‘Human Extracellular Matrix and Adhesion Molecules’ RT2 Profiler PCR Arrays (Qiagen) were used. These RT2 Arrays profile simultaneously the expression of 84 genes involved in cell-cell contact and cell adhesion, within one sample. In brief, cells were harvested and mRNA was isolated using the ReliaPrep^TM^ RNA Cell Miniprep System (Promega). The mRNA concentration was measured with a NanoDrop Fluorospectrometer (Thermo Fisher Scientific, Inc.; Waltham, MA, USA). For cDNA synthesis the RT2 First Strand Kit was used. A total of 0.5 µg RNA was reverse transcribed and the cDNA was amplified by the RT2 SYBR Green Master mix. Data were evaluated using the Qiagen PCR Array Data Analysis Web portal. Gene expression was normalized to the mean expression of the reference genes (GAPDH, ACTB, B2M HPRT and RPLP0) and was calculated using the ∆∆CT method.

### siRNA-mediated knockdown

For siRNA-mediated knockdown, cells were seeded in 6-well plates and were grown overnight to 20% confluence. For transfection, a total of 100 pM siRNA (ON-Target plus Smart pools, Dharmacon; for double knockdown 50 pM of siRNA for target 1 and for target 2), and 5 μl of Lipofectamine RNAiMAX were mixed with Opti-MEM I and incubated for 20 min at room temperature. Then the formed siRNA/lipid complexes were carefully added per well (to a final concentration of 50 nM siRNA). Cells were incubated for 72 hours and grown under normal conditions to 90% confluence. Subsequently, the transfected cells were used for spheroid formation and Western blotting.

### Western blotting

For Western blotting whole cell lysates were prepared from spheroids. Briefly, spheroids were washed in PBS and extracted in RIPA lysis buffer (50 mM Tris pH 7.6, 150 mM NaCl, 1% TritonX-100, 0.1% SDS, 0.5% sodium deoxycholate, 1 mM PMSF, 4 µg/ml aprotinin, 4 µg/ml leupeptin, 0.6 µg/ml benzamidinchloride, 20 µg/ml trypsin inhibitor). Samples (10–15 µg of total protein content) were mixed with 4 × loading dye (200 mM Tris pH 6.8, 400 mM DTT, 8% SDS, 0.4% bromophenol blue, 40% glycerol), denatured at 95 °C for 5 min, subjected to SDS-PAGE and transferred to a nitrocellulose membrane. Blocked membranes were probed with primary antibodies at 4 °C overnight and with horseradish peroxidase-conjugated secondary antibodies (anti-mouse IgG-heavy and light chain antibody and anti-rabbit IgG-heavy and light chain antibody, Bethyl Laboratories Inc., Montgomery, TX) at RT for 1 h. Signals were detected using enhanced chemiluminescence. Primary Antibodies used were: anti-E-Cadherin (clone HECD1, mouse monoclonal, 1:1000, Abcam ab1416), anti-P-Cadherin (clone OTI2D5, mouse monoclonal, 1:4000, OriGene TA506403), anti α-Catenin (clone 1G5, mouse monoclonal, 1:500, Thermo Scientific MA1-2000), anti GAPDH (rabbit, 1:10000, Trevigen 2275-PC-100), anti α-Tubulin (clone DM1A, mouse monoclonal, 1:1000, Merck-Millipore, Calbiochem CP06).

### Immunofluorescence

Immunofluorescence staining was performed on whole mount spheroids as described^54^. In brief, spheroids were fixed in PBS containing 4% PFA and 1% Triton X-100 for 3 h at 4 °C and washed in PBS. Spheroids were then dehydrated in an ascending series of methanol and subsequently rehydrated. After blocking in PBST (0.1% Triton X-100 in PBS)/3% Bovine Serum Albumin (MP Biomedicals, Illkirch, France) o/n at 4 °C and washing in PBST spheroids were incubated with primary antibodies in PBST at 4 °C for 48 h and washed followed by incubation with Alexa Fluor conjugated secondary antibodies for 24 h. Cell nuclei were counterstained with DAPI. 5 µm optical sections were recorded on a on a Leica-SP8 with a 20× immersion objective (NA = 1.3; Leica Microsystems, Wetzlar, Germany). Antibodies used were: E-cadherin (HECD-1, ab1416, Abcam, 1:150), P-Cadherin (TA506403, OriGene, 1:100), EpCAM (ab8601, Abcam, 1:50), phalloidin-Alexa Fluor 594 (A 22283, Thermo Scientific, Waltham, MA, 1:40). Anti-mouse-IgG-heavy-and-light-chain (Bethyl Laboratories Inc., Montgomery, TX) was used as IgG control. Alexa Fluor 488-conjugated secondary antibody (Thermo Scientific) was used at 1:500.

### Migration and invasion assays

For migration 2.5 × 10^4^ cells were seeded in serum-free medium in the upper chamber of transwell migration inserts (8 µm, Sigma-Aldrich). The cells were allowed to migrate towards complete medium. After 20 hours, non-migrated cells were removed and migrated cells were fixed (Roti-Histofix 4%) and stained for 30 min with 0.006% (w/v) crystal violet-solution (Sigma-Aldrich). Five random images were taken and membrane coverage was determined (ImageJ64) or cells were counted manually. The invasion assay was performed similar to the migration assay except that the transwell inserts were prior covered with collagen I gel and the experiment was analyzed after 24 hours. For collagen gel invasion assays 200 single cells were embedded in Collagen I gel as described^55^. The medium was changed every second day. After 6–13 days pictures of the formed colonies were taken.

### Statistical analysis

Quantitative experiments were conducted at least in biological triplicates and statistical significance was calculated using the Student’s T-test or one way ANOVA. P-values are indicated. The number of replicates is always indicated in the Figure legends.

## Electronic supplementary material

Supplemental Figures

Supplementary Dataset
