# Supplementary material for: Exclusion from spheroid formation identifies loss of essential cell-cell adhesion molecules in colon cancer cells
Source: Sci Rep. 2018 Jan 18;8:1151. doi: 10.1038/s41598-018-19384-0 (PMC5773514; doi:10.1038/s41598-018-19384-0)
Supplement: Supplementary file 1 — Supplemental Figures [file 41598_2018_19384_MOESM1_ESM.pdf]

Supplemental Figures 1-8 to:

Exclusion from spheroid formation identifies loss of essential cell-cell adhesion molecules in colon cancer cells

Mira Stadler, Martin Scherzer, Stefanie Walter, Silvio Holzner, Karoline Pudelko, Angelika Riedl, Christine Unger, Nina Kramer, Beatrix Weil, Jürgen Neesen, Markus Hengstschläger and Helmut Dolznig

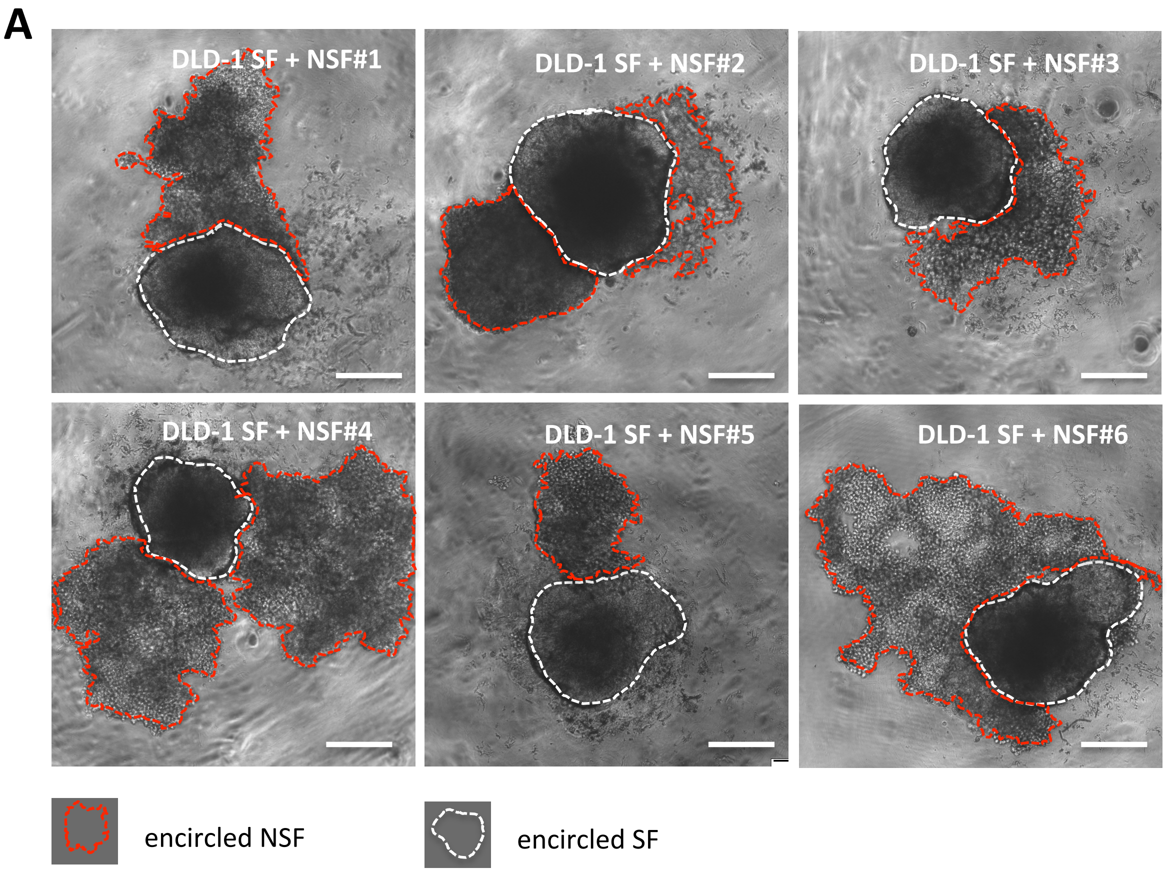

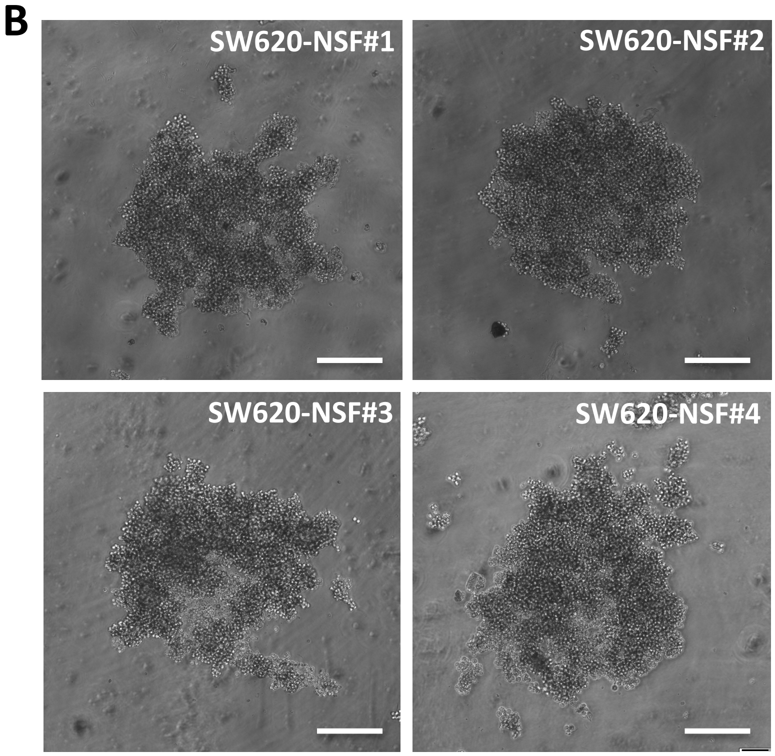

Supplemental Figure S1: *Separation of SF and NSF cells in single 96 wells* **A.** DLD-1 spheroids were gener-ated in ULA 96 well plates and cultured for 14 days with partial medium changes every third day. Representative phase contrast images are shown. Spheroids are encircled in white while NSF cells are surrounded by a red dotted line. Scale bars: 100 µm. **B** Different SW620-NSF clones generated at different time points display the same phenotype. Representative phase contrast pictures of 4 different NSF cultures are shown. Scale bars: 100 µm.

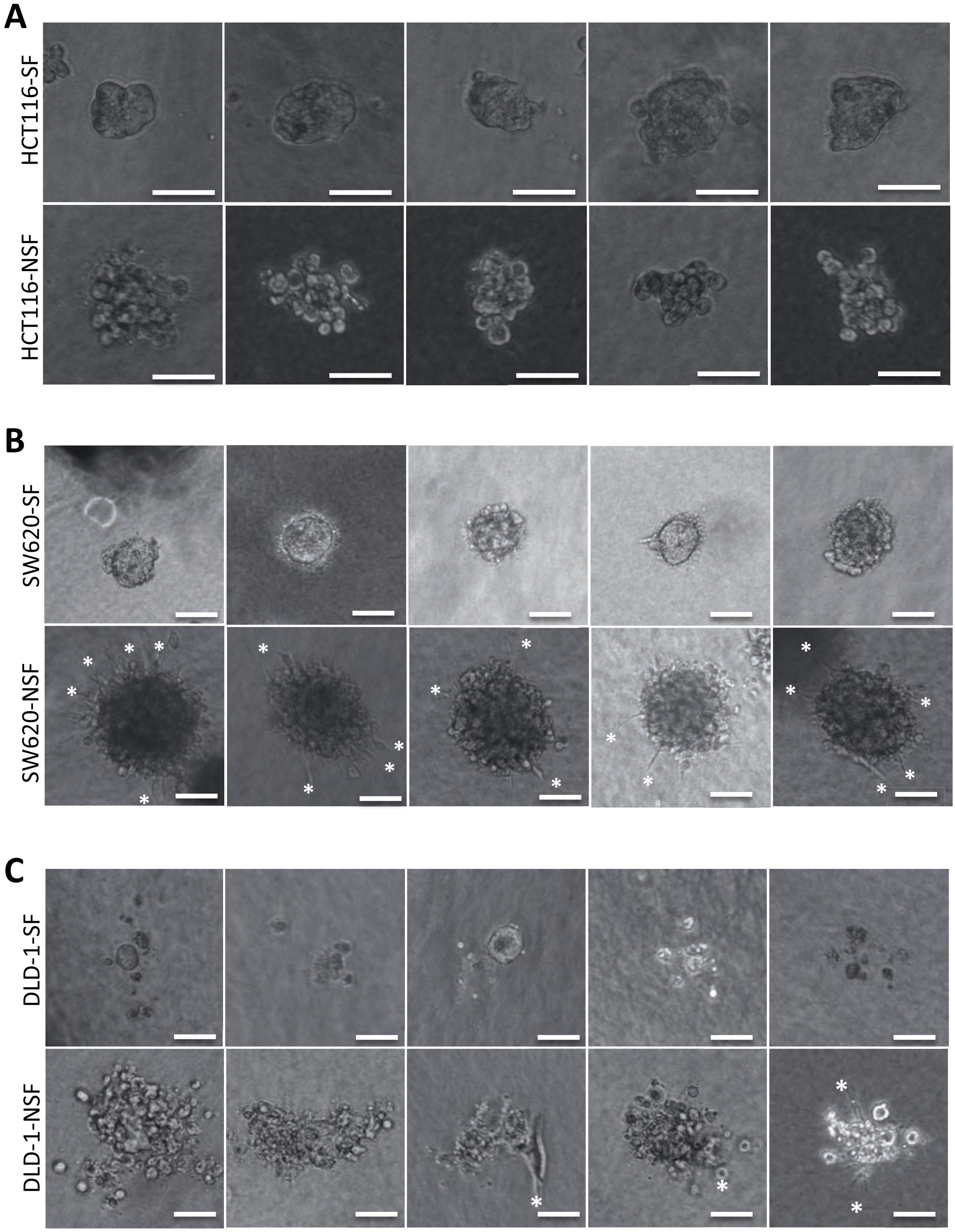

Supplemental Figure S2*: Single cell derived colonies in collagen I gels.* **A** Phase contrast images of HCT116-SF and NSF cells after 6 days in collagen I gel. **B** Phase contrast images s of SW620-SF and NSF cells after 6 days in collagen I gel. Stars indicate spindle like cells invading into the matrix. . **C** Phase contrast images of DLD-1-SF and NSF cells after 6 days in collagen I gel. Stars indicate spindle like cells invading into the matrix. Scale bars: 50 µm.

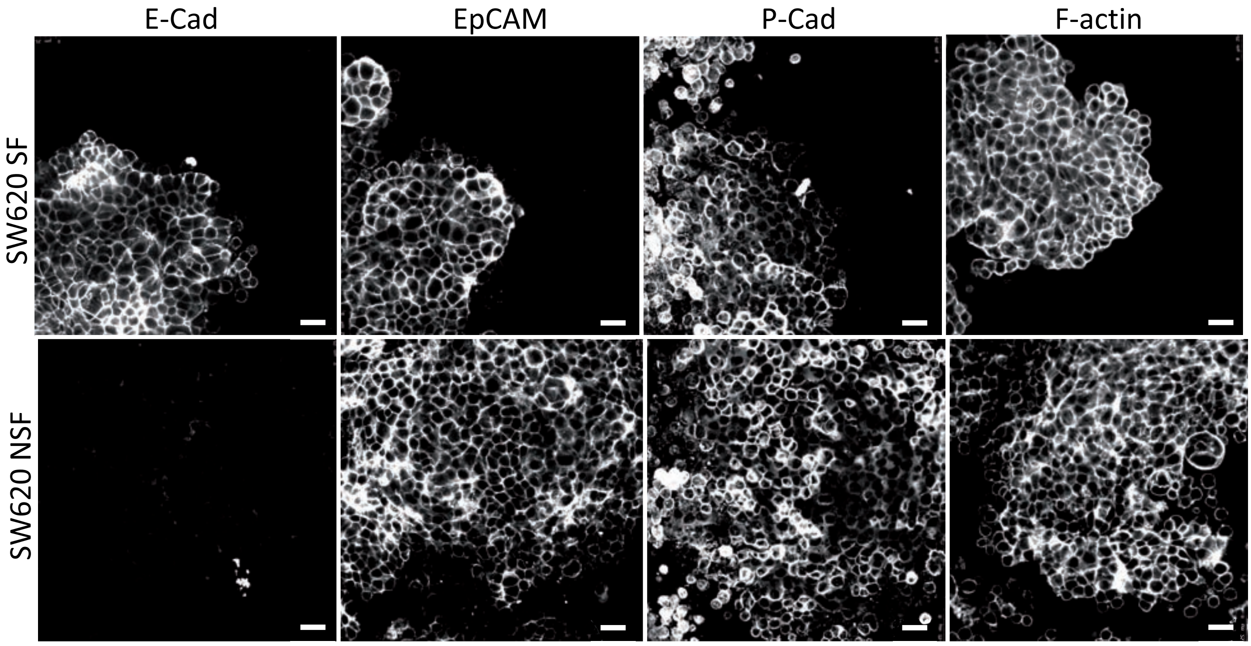

Supplemental Figure S3*:* IF analysis of E-cadherin, EpCAM, P-cadherin and F-actin expression in SW620-SF versus SW620-NSF cells. These images correspond to the images shown in Figure 6A and are single colored for better evaluation of staining intensities and distribution of the analyzed adhesion markers. Scale bars: 25 µm.

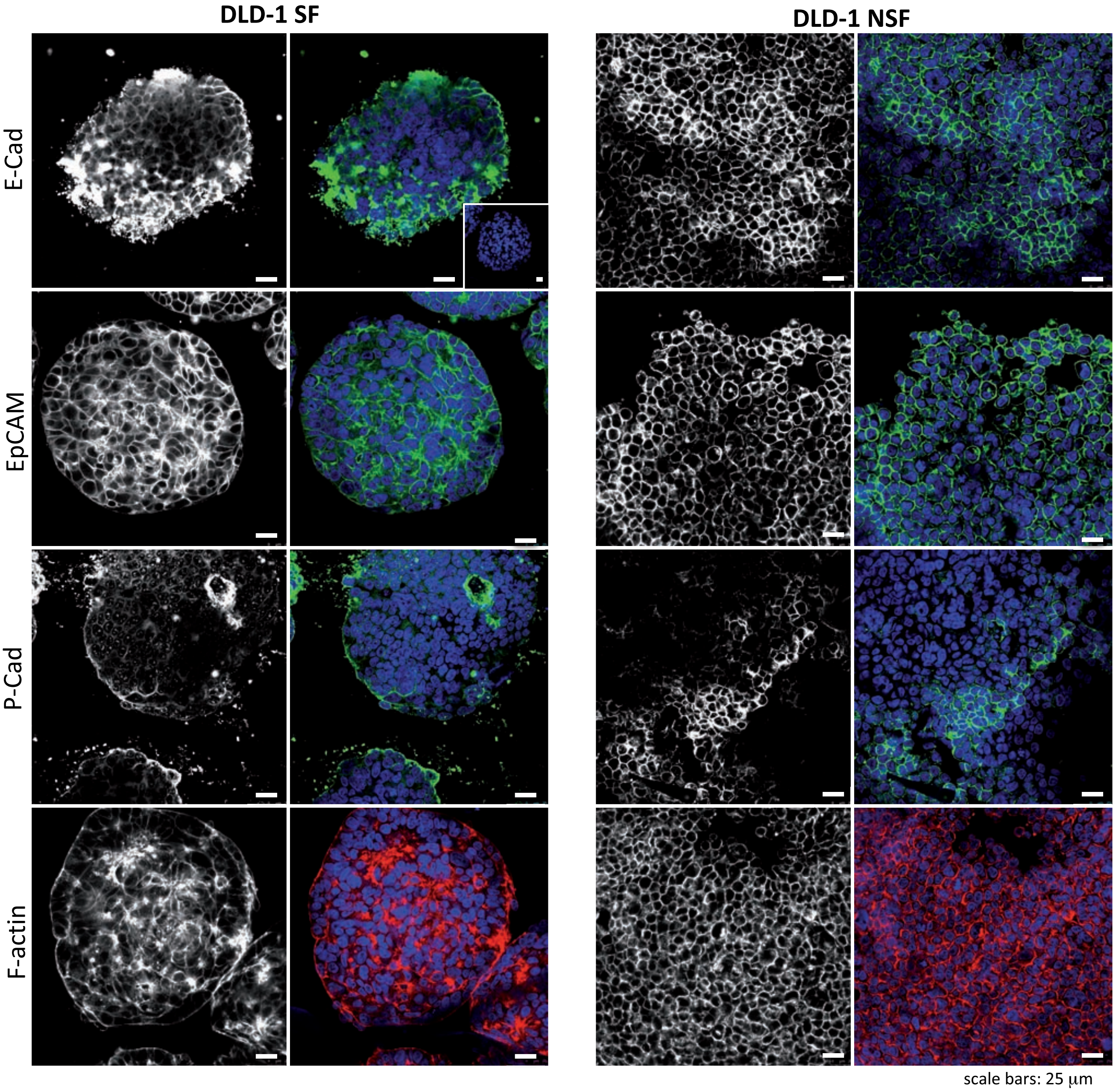

Supplemental Figure S4*:* IF analysis of E-cadherin, EpCAM, P-cadherin (all green) and F-actin (red) expression in HCT116-SF and HCT116-NSF spheroids or cell assemblages. Cell nuclei are stained with DAPI (blue). Small inset: IgG control. Scale bars: 25 µm. For better assessment of staining intensities and distribution the analyzed adhesion markers are also shown in single colored images (black and white). Scale bars: 25 µm.

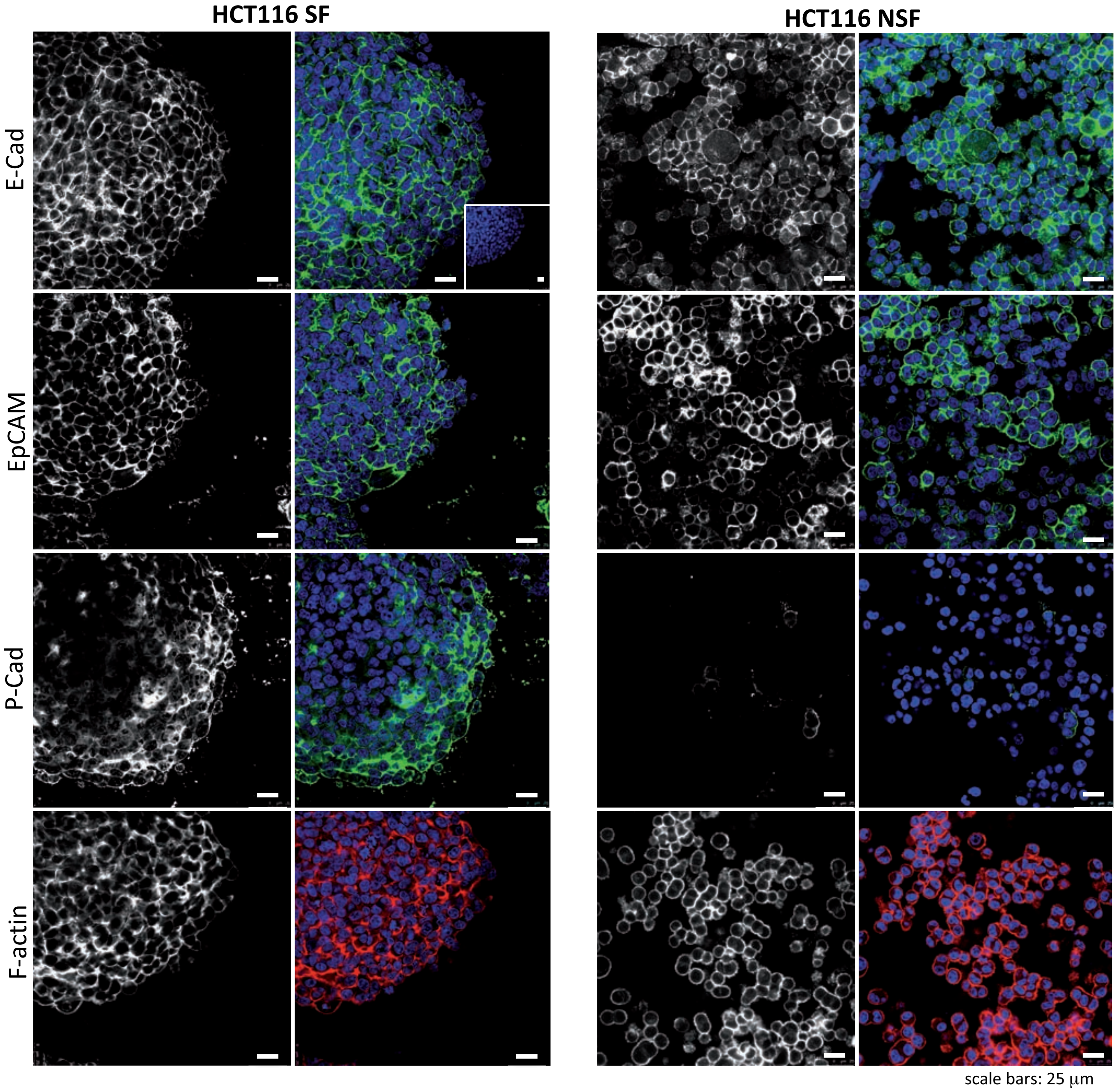

Supplemental Figure S5*:* IF analysis of E-cadherin, EpCAM, P-cadherin (all green) and F-actin (red) expression in DLD-1-SF and DLD-1-NSF spheroids or cell assemblages. Cell nuclei are stained with DAPI (blue). Small inset: IgG control. Scale bars: 25 µm. For better assessment of staining intensities and distribution the analyzed adhesion markers are also shown in single colored images (black and white). Scale bars: 25 µm.

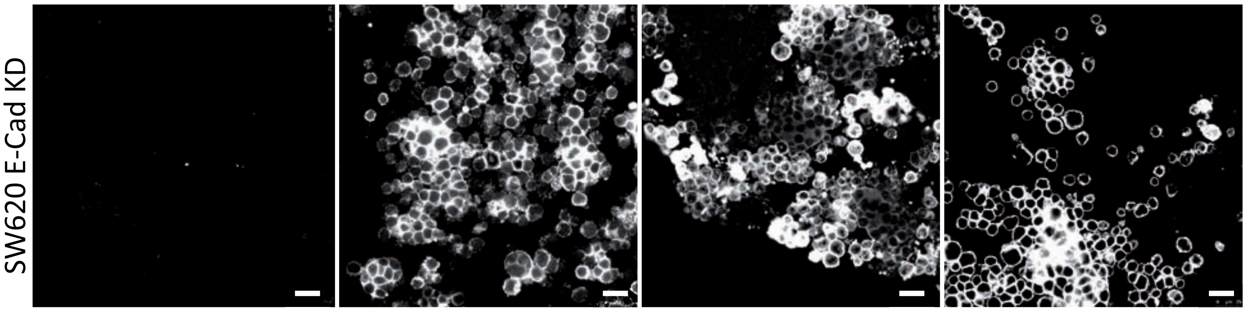

Supplemental Figure S6*:* IF analysis of E-cadherin, EpCAM, P-cadherin and F-actin expression in SW620-NTC versus SW620-CDH1-KD cells. These images correspond to the images shown in Figure 6C and are single colored for better evaluation of staining intensities and distribution of the analyzed adhesion markers. Scale bars: 25 µm.

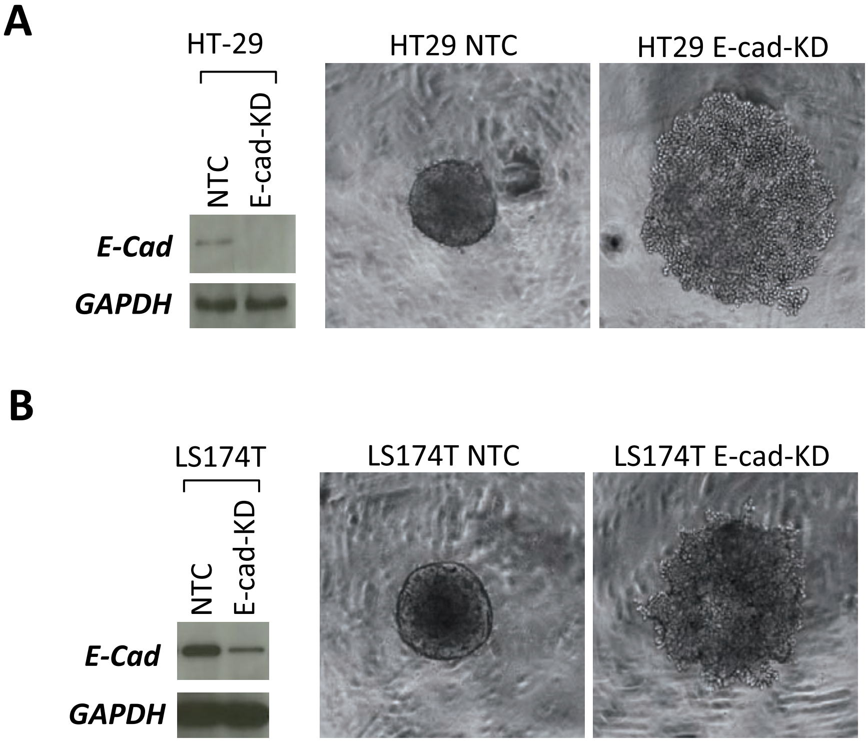

Supplemental Figure S7: *E-cadherin knockdown in HT29 and LS174T induce loss of spheroid formation.* **A** HT-29 and **B** LS174T cells were transfected with non targeting control (NTC) or CDH1 (E-cadherin) targeting siRNAs and subsequently subjected to spheroid formation in ULA 96 well plates. Knockdown efficiency was tested by Western blotting (left panels). Representative images of cells after two days in the wells are shown (right panels).

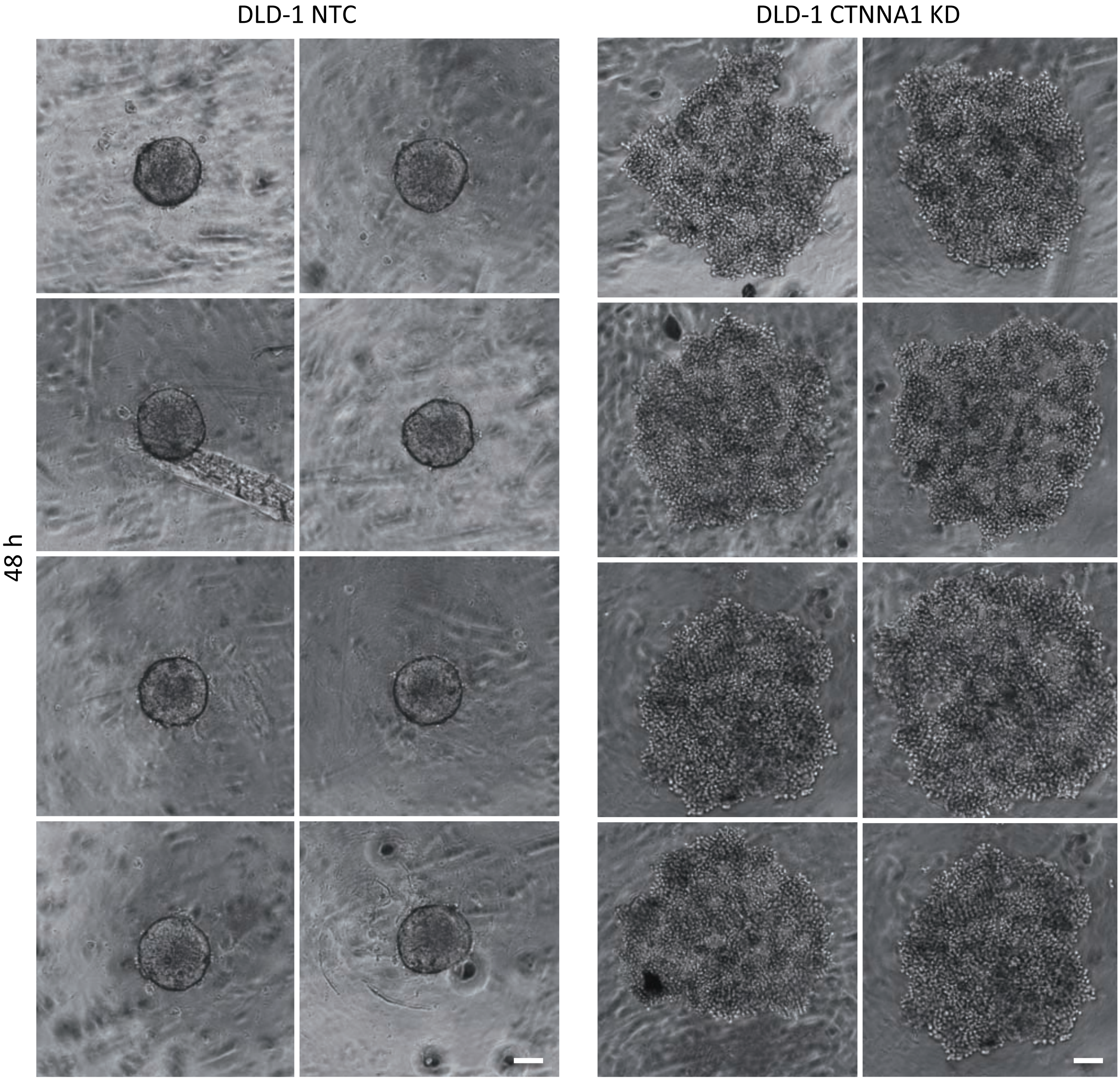

Supplemental Figure S8: *CTNNA1 (alpha catenin) knockdown in DLD-1 cell induce loss of spheroid formation.* DLD-1 cells were transfected with non targeting control (NTC) or CTNNA1 (alpha catenin) targeting siRNAs and subsequently subjected to spheroid formation in ULA 96 well plates. Representative images of eight different wells per condition after two days of culture are shown.
